# Accuracy of a Deep Learning Model in Intracardiac Echocardiography

**DOI:** 10.1016/j.jacadv.2026.102867

**Published:** 2026-06-15

**Authors:** Devi Nair, Jeffrey Winterfield, Jonathan C. Hsu, Rakesh Gopinathannair, Larry Chinitz, Naga Venkata K. Pothineni, Frederick T. Han, Bishnu P. Dhakal, Chirag Barbhaiya, Amish S. Dave, Fermin Garcia, Matthew C. Hyman, Travis Dahlen, Kristi Tanouye, Jason Yao, Shubhadarshini Pawar, Judith Buckland, Anna Gilgur, Raphael Elspas, Aakriti Gupta, Roman A. Sandler, Joseph Z. Sokol

**Affiliations:** a St. Bernards Heart & Vascular Center, Jonesboro, Arkansas, USA; b Medical University of South Carolina (MUSC), Charleston, South Carolina, USA; c University of California San Diego (UCSD), La Jolla, California, USA; d Kansas City Heart Rhythm Institute, Kansas City, Missouri, USA; e NYU Langone Health, New York, New York, USA; f Houston Methodist Hospital, Houston, Texas, USA; g University of Pennsylvania, Philadelphia, Pennsylvania, USA; h Abbott Laboratories, Chicago, Illinois, USA; i CardioServ, Boynton Beach, Florida, USA; j Karsh Center for Interventional Cardiology, Smidt Heart Institute, Cedars-Sinai Medical Center, Los Angeles, California, USA; k iCardio.ai Corporation, Los Angeles, California, USA

**Keywords:** atrial fibrillation, catheter ablation, deep learning, electrophysiology, intracardiac echocardiography, intraprocedural imaging, semantic segmentation

## Abstract

**Background:**

Intracardiac echocardiography (ICE) is widely used during electrophysiology and structural heart procedures; however, image interpretation remains operator-dependent and procedural views are not standardized. Although artificial intelligence has been increasingly applied to transthoracic and transesophageal echocardiography, applications to ICE remain limited.

**Objectives:**

The objective of the study was to develop and evaluate Auto-Contour, a deep-learning pipeline for multistructure semantic segmentation of ICE anatomy and assess its feasibility for real-time procedural guidance.

**Methods:**

In this retrospective multicenter study, 5,496 deidentified ICE cine loops from 249 procedures of unique patients, including routine clinical cases and the ViewFlex™ X first-in-human study, were analyzed. ICE experts classified each cine into 1 of 20 procedural views and annotated key anatomic structures, including the left atrium, left atrial appendage, pulmonary vein ostia, valves, cusps, papillary muscles, and left ventricle, at end-systole, and end-diastole, yielding 65,117 segmentations. A deep-learning segmentation model was trained using patient-level splits, standard augmentations, and early stopping.

**Results:**

Segmentation performance was highest for larger cardiac chambers, with Dice scores of 0.94 for the left atrium and 0.82 for the left ventricle, and corresponding 95th-percentile Hausdorff distance values of 1.18 mm and 3.27 mm. Smaller structures also demonstrated acceptable performance, including the left atrial appendage, pulmonary veins, papillary muscles, and aortic cusps. The mean per-frame inference time was <0.03 seconds.

**Conclusions:**

Auto-Contour demonstrated robust multistructure segmentation of ICE anatomy with real-time inference, supporting prospective evaluation of artificial intelligence-assisted ICE for procedural standardization, efficiency, and safety.

Intracardiac echocardiography (ICE) is an imaging modality that provides real-time, high-resolution ultrasound views from within the heart, making it invaluable in interventional electrophysiology and structural heart procedures.^1^^,^^2^ ICE is used in catheter ablation in the atria and ventricles for arrhythmias, such as atrial fibrillation and ventricular tachycardia, and for transseptal puncture guidance, catheter visualization, and structure identification.^3^^,^^4^ It is also used for structural interventions such as left atrial appendage occlusion, septal defect closure, and prosthetic valve implantations.^5^ The key advantages of ICE include real-time visualization of cardiac structures, reducing reliance on fluoroscopy and associated radiation, and often obviating the need for general anesthesia, as ICE can be used with the patient under conscious sedation.^6^^,^^7^ Using ICE during complex ablations has been associated with improved procedural safety and outcomes, with studies showing that incorporating ICE in ventricular tachycardia ablation was linked to a significantly lower likelihood of arrhythmia recurrence and repeat ablations at 12-month follow-up.^8^ The evidence toward the benefit of ICE points to its essential role in interventional electrophysiology and structural heart interventional procedures.

Despite its advantages, effective use of ICE is highly operator dependent. Achieving and maintaining optimal views requires considerable expertise in both catheter manipulation and image interpretation.^8^ Unlike standard transthoracic or transesophageal echocardiography, which has well-defined views, ICE imaging has limited standardized views and often demands the operator to mentally navigate 3-dimensional cardiac anatomy from within their respective 2-dimensional images.^8^^,^^9^ This can result in challenges acquiring and interpreting ICE images, leading to variability in procedure quality, consistent with the evidence that intraoperative imaging experience is tied to procedural outcomes. Therefore, a technology that can assist, automate, and standardize ICE interpretation may lead to procedural consistency and improve outcomes.^10^^,^^11^

Meanwhile, artificial intelligence (AI) has matured in recent years, with clinical practice embracing the utility of AI and its impact on clinical workflow.^12^^,^^13^ Prior AI work in echocardiography has focused primarily on transthoracic and transesophageal imaging; applications to ICE remain largely unexplored, limited only to anatomy identification and guidance.^14^^,^^15^ To the best of our knowledge at the time of manuscript preparation, this work represents the earliest comprehensive application of deep learning to anatomical reconstruction from ICE segmentations. Partly owing is the paucity of large data sets of ICE studies, which are not routinely saved in clinical practice. Moreover, because acquisition of ICE requires the coordinated effort of multiple personnel and systems, developing this data set demands extensive logistical collaboration across a broad network of organizations.

Auto-Contour is an automated semantic segmentation pipeline for ICE images designed to automatically label cardiac anatomical structures on 2-dimensional ICE images. These labels supplement the manual intraoperative annotation process used to obtain contours that, together with integrated catheter position and orientation data, can be used to build 3-dimensional anatomical projections. The use of an AI model to augment the 3-dimensional anatomical modeling workflow may improve procedural consistency, accuracy, and speed by highlighting anatomy, including cardiac chambers and pulmonary vein ostia, on the integrated ICE 3-dimensional mapping system, thereby assisting with procedural navigation and catheter positioning.

The aim of this study was to evaluate the accuracy of Auto-Contour for ICE image segmentation by determining how well an AI model trained on expertly annotated ICE images can identify and outline key cardiac structures. It was hypothesized that the model could achieve high agreement with expert manual contours, as measured by the Dice similarity coefficient and Hausdorff distance (HD), across a variety of ICE views and anatomical targets. Success in this endeavor would provide a foundation for real-time AI assistance during procedures, improving the accuracy, speed, and consistency of ICE usage, and paving the way for integration with 3-dimensional mapping systems for advanced image-guided therapy.

## Methods

### Study design and data sources

We performed a retrospective analysis of ICE imaging from 2 sources collected between June 2024 and December 2025: 1) routine clinical ICE acquired with the Abbott ViewFlex catheter and ViewMate console during real-world electrophysiology procedures; and 2) the ViewFlex X First-in-Human study (NCT06772493), which evaluated a sensor-enabled ICE catheter (ViewFlex X ICE Catheter, Sensor Enabled, Abbott) integrated with the EnSite X mapping system. The protocol conformed to the Declaration of Helsinki and was approved by the institutional review boards of participating centers. Given the retrospective design, the requirement for informed consent was waived.

Across participating high-volume electrophysiology centers, the aggregated data set comprised 5,496 deidentified ICE Digital Imaging and Communications in Medicine (DICOM) cine loops from 249 procedures of unique patients (binned median age 65-69 [IQR: 55-59 to 70-74], 36% female, with common comorbidities including hypertension, diabetes, and atrial fibrillation). Of the total cine data set, approximately 65% originated from routine clinical ICE studies and 35% from the first-in-human trial, ensuring representation across diverse imaging sources and catheter platforms. Cine loops that contained catheters in view were excluded. Frames representing systolic and diastolic points in the cardiac cycle were annotated with the addition of intracycle annotations to increase the quantity of segmentations for features less represented, such as the pulmonary veins. Left atrial anatomical targets were imaged with the ICE catheter positioned in the right atrium and, in some patients, following transseptal puncture, from within the left atrium itself. Left ventricular targets were obtained from the right ventricle. A dozen ICE experts solicited for collecting annotations had a minimum of 5 years of experience with intraprocedural imaging.

### Image annotation and ground truth

We defined a standardized set of 20 procedural ICE views reflecting typical orientations and catheter positions during left atrial ablation and related procedures. Examples included the right-atrial “home” view, interatrial septum view for transseptal puncture, left atrial view, ostial views for each of the 4 pulmonary veins, the left atrial appendage view (often from the pulmonary artery or left atrial ridge), mitral valve view, aortic valve view, coronary sinus view, and right ventricular outflow tract.

**Figure 1:**
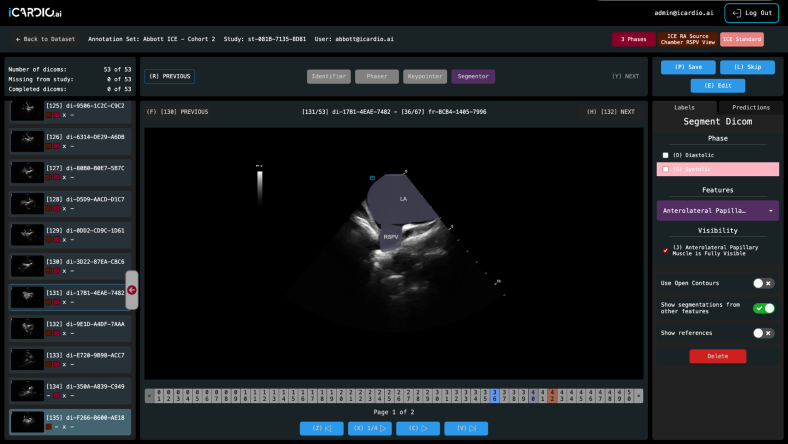
Expert Annotation Workflow Using the iCardio.ai Labeling Platform Representative example of the iCardio.ai labeling platform using intracardiac echocardiography data from the Abbott First-in-Human study. The displayed ICE frame shows expert annotation of the left atrial body and right superior pulmonary vein. Additional cine frames can be selected and annotated to generate expert-derived ground truth labels for model training and evaluation.

Annotations were performed in iCardio.ai’s internal browser-based Labeling Platform (Figure 1). Each cine was reviewed by ICE experts, assigned a single view label from the 20-view ontology, and manually annotated by tracing contours on representative frames (typically 1 to 3 frames per cine at end-systole or the phase with best visualization). Labeled structures included the left atrium, left atrial appendage, pulmonary vein ostia (right superior, right inferior, left superior, left inferior, and right and left common vein), mitral valve, aortic valve, noncoronary, right coronary, left coronary cusps, and left ventricle. For training, we sampled at least 2 frames per cine loop at end-systole and end-diastole or best visualization, with intracardiac cycle sampling for pulmonary veins; on average, 5 frames per cine were used. All annotations were cross-checked by a second expert, with disagreements adjudicated by a senior annotator. In total, 65,117 individual anatomic segmentations were accumulated. On average, each cine contained multiple labeled structures (eg, both left atrial and left atrial appendage in a left atrial appendage view), reflecting the complexity of ICE imaging.

### Model architecture

Our segmentation approach employs a U-Net–style semantic segmentation network that produces pixel-wise class predictions for relevant anatomy pretrained on a transthoracic echocardiographic image segmentation task. Input images are resized to 256×256 pixels before being fed into the network. The model is trained using a Generalized Dice Focal Loss.^16^ The model is optimized using AdamW with an initial learning rate of 0.001, weight decay of 1 × 10^−5^.

### Training, validation, and tuning

Data were partitioned on the patient level (approximately 80% training, 20% validation/testing) such that no patient, cine loop, or annotated frame contributing to the validation cohort was available to the model during training. Hyperparameters, including learning rate, network depth, batch size, and augmentation intensity, were tuned empirically through systematic validation set evaluation. To mitigate overfitting, we employed early stopping with model checkpointing based on the best validation Dice score, terminating training if no improvement was observed.

### Evaluation metrics

Dice and HDs were calculated between Auto-Contour and expert reference masks for each anatomic structure. Because the analytic data set included multiple annotated frames from the same cine loop and multiple structure instances within a given study, observations were clustered rather than statistically independent. Accordingly, Dice and 95th-percentile Hausdorff distance (HD95) were summarized descriptively at the structure-instance level, with patient-level partitioning used to prevent overlap between development and validation cohorts. Because Dice captures spatial overlap but may not fully reflect qualitative operator preference, it was used primarily as the quantitative development metric. HD was computed as the 95th percentile bidirectional surface distance between predicted and expert contours, reported in millimeters using DICOM pixel spacing, and selected as a stricter assessment of contour alignment. In addition, per-image processing time (s) was qualitatively described as near-real-time as per feedback from users, pointing to feasibility for real-time clinical use. All analyses were performed using Python and MATLAB.

### TRIPOD + AI reporting guideline

This study was reported in accordance with the TRIPOD + AI reporting guideline, as applicable to machine-learning–based image segmentation studies. A completed TRIPOD + AI checklist is provided in the Supplemental Appendix.

## Results

A total of 5,496 deidentified ICE DICOM cine loops from 249 electrophysiology procedures were collected across multiple high-volume centers, representing a variety of atrial and ventricular arrhythmia ablations as well as other electrophysiology interventions. The distribution of procedural indications is summarized in Table 1. All imaging data were deidentified and analyzed under institutional review board–approved protocols. The overall study design, Auto-Contour workflow, segmentation outputs, and potential clinical applications are summarized in the Central Illustration.

**Table 1:** Baseline Characteristics and Procedural Indications of the Auto-Contour Study Cohort

Total studies/procedures	249 (100)
Age, median (IQR), years	67 (57-72)
Sex	
Men	154 (61.8)
Women	83 (33.3)
Unknown	12 (4.8)
Race/ethnicity	
White	213 (85.5)
Black or African American	15 (6.0)
Hispanic or Latino	1 (0.4)
Asian	3 (1.2)
Native Hawaiian or other Pacific Islander	1 (0.4)
Other	1 (0.4)
Missing/unavailable	15 (6.0)
Procedural indication	
Atrial arrhythmias	213 (86)
Paroxysmal atrial fibrillation	135 (54)
Persistent atrial fibrillation	59 (24)
Atrial flutter	10 (4)
Supraventricular tachycardia	9 (4)
Ventricular arrhythmias	33 (13)
Premature ventricular contractions	24 (10)
Ventricular tachycardia	9 (4)
Other EP procedures	3 (1)

Percentages are expressed as proportion of the total 249 procedures included in the data set.

EP = electrophysiology.

**Figure 2:**
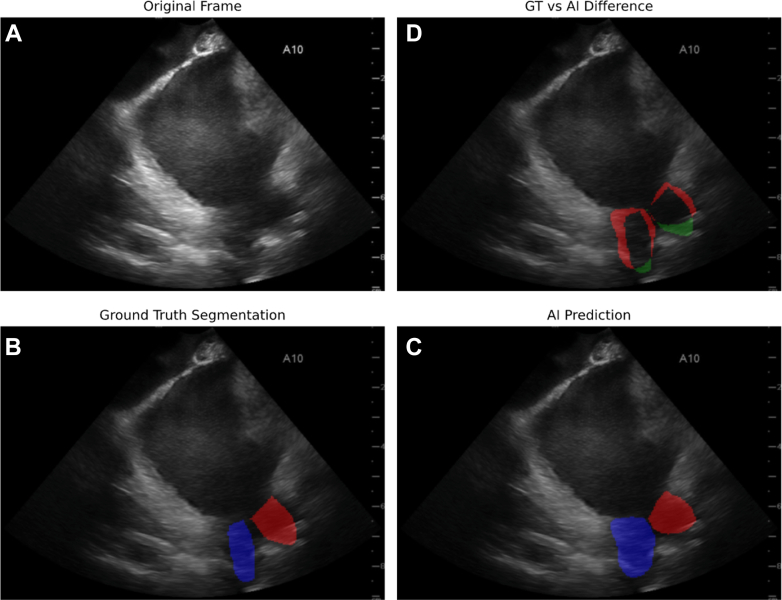
Auto-Contour vs Expert Segmentation of the Left Pulmonary Veins Representative intracardiac echocardiography data from the Abbott First-in-Human study. (A) Original ICE frame acquired with the ViewFlex X catheter in a left pulmonary vein view. (B) Expert ground truth segmentation of the left inferior pulmonary vein and left superior pulmonary vein. (C) Auto-Contour–generated segmentation of the same ICE frame. (D) Overlay comparison of expert and Auto-Contour segmentations demonstrating close correspondence in pulmonary vein boundary delineation. Red indicates areas where the Auto-Contour boundary extends beyond the expert annotation, and green indicates areas where the expert annotation extends beyond the Auto-Contour boundary. AI = artificial intelligence; GT = ground truth.

**Figure 3:**
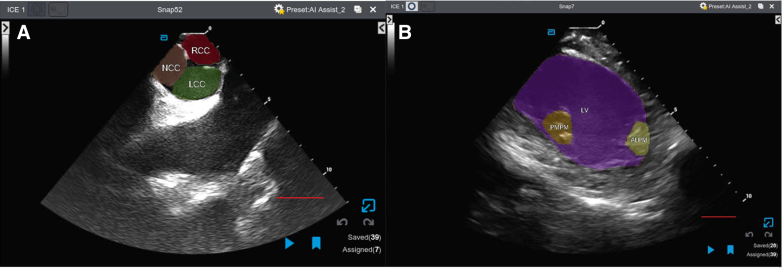
Representative Auto-Contour Segmentations of Valvular and Left Ventricular Structures Selected examples from the Abbott First-in-Human study demonstrating Auto-Contour–generated intracardiac echocardiography segmentations of (A) the aortic cusps, including the right coronary cusp, left coronary cusp, and noncoronary cusp and (B) left ventricular structures, including the left ventricle, anterolateral papillary muscle, and posteromedial papillary muscle. ALPM = anterolateral papillary muscle; LCC = left coronary cusp; LV = left ventricle; NCC = noncoronary cusp; PMPM = posteromedial papillary muscle; RCC = right coronary cusp.

**Figure 4:**
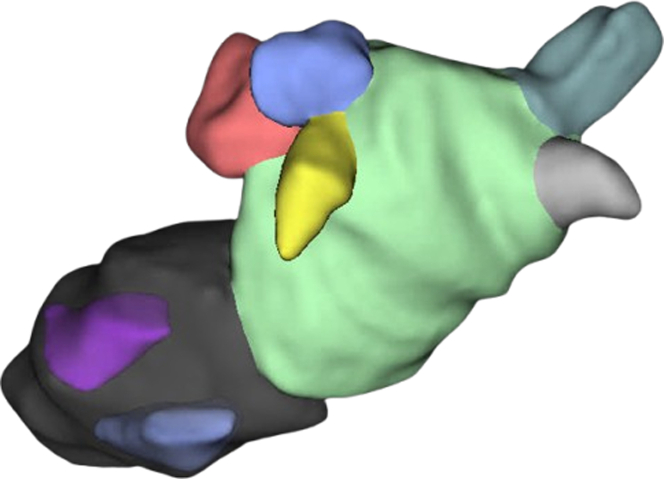
3-Dimensional Surface Reconstruction From Auto-Contour–Predicted Segmentation Contours Representative example from the Abbott First-in-Human study showing 3-dimensional surface reconstruction of the left atrium and left ventricle in the EnSite X Mapping System. Auto-Contour–predicted intracardiac echocardiography segmentation contours were projected into 3-dimensional space using position and orientation data from the magnetic sensor in the ViewFlex X Ultrasound Catheter.

**Figure 5:**
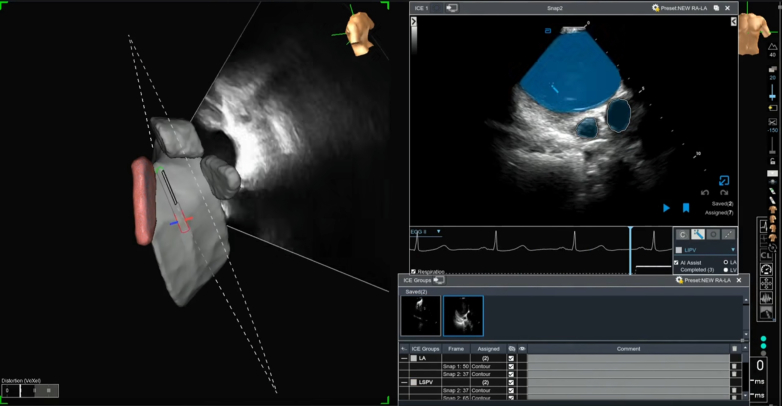
Rendering of Left Atrium Body Surface in Abbott’s EnSite System from Auto-Contour-Predicted Segmentations Representative Abbott EnSite™ X Mapping System interface demonstrating 3-dimensional reconstruction of the left atrial body surface using Auto-Contour–predicted segmentations from intracardiac echocardiography. The ICE Reveal fan is displayed within the 3-dimensional map to demonstrate spatial correspondence between the reconstructed anatomy and the intracardiac echocardiography image. The saved ICE frame shows AI-assisted contour segmentation of the left atrium and adjacent left atrial structures.

**Figure 6:**
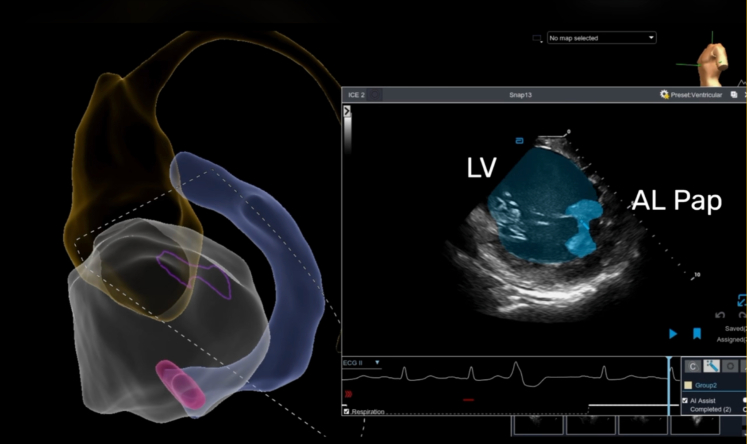
Left Ventricular Surface Reconstruction in the EnSite X Mapping System From Auto-Contour–Predicted Segmentations EnSite X Mapping System example interface showing a 3-dimensional reconstruction of a left ventricle, aorta, and coronary sinus. A saved ICE frame is shown depicting Auto Contour-predicted left ventricle and anterior lateral papillary muscle with the location of the ICE frame shown as a dotted-line in 3-dimensional space. Data from Abbott’s First in Human study. ICE = intracardiac echocardiography; other abbreviations as in Figure 3.

**Central Illustration:**
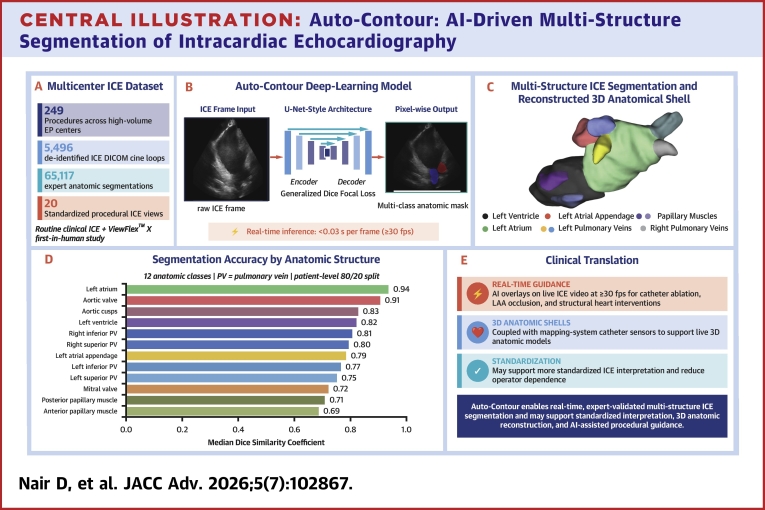
Auto-Contour: AI-Driven Multi-Structure Segmentation of Intracardiac Echocardiography Auto-Contour is a deep-learning model for real-time, multiclass anatomic segmentation of intracardiac echocardiography (ICE) images. (A) Multicenter ICE data set of 249 procedures, 5,496 deidentified ICE Digital Imaging and Communications in Medicine (DICOM) cine loops, 65,117 expert segmentations, and 20 standardized procedural ICE views. (B) Raw ICE frames are processed through a U-Net–style architecture to generate pixel-wise segmentation masks in real time (<0.03 seconds/frame; ≥30 frames/s). (C) Representative multistructure segmentation and reconstructed 3-dimensional anatomic shell. (D) Segmentation performance across 12 anatomic classes, with median Dice similarity coefficients ranging from 0.69 to 0.94 and median 95th percentile Hausdorff distance (HD95) ranging from 0.81 to 3.27 mm. (E) Potential applications include real-time image overlay, live 3-dimensional anatomic modeling, and standardized ICE interpretation. AI = artificial intelligence; 3D = 3-dimensional.

### Segmentation accuracy

The model demonstrated strong segmentation performance across major cardiac structures, with detailed results provided in Table 2. Left atrial chamber segmentation achieved a median Dice coefficient of 0.94, reflecting high agreement with expert contours. In practical terms, a Dice of 0.94 indicates excellent agreement between Auto-Contour and expert annotations, comparable to leading segmentation results reported in other modalities such as cardiac magnetic resonance imaging.^17^ Left ventricle chamber segmentation achieved a median Dice of 0.82.

**Table 2:** Anatomical Region Counts and Segmentation Performance by Structure

Class/Region	N	Dice	HD95 (mm)
Left atrium	1,981	0.937 [0.906-0.954]	1.18 [0.79-1.92]
Left atrial appendage	410	0.785 [0.659-0.852]	1.81 [1.07-3.27]
Left inferior pulmonary vein	410	0.766 [0.666-0.829]	1.56 [1.04-2.71]
Left superior pulmonary vein	347	0.752 [0.625-0.831]	1.49 [0.86-2.76]
Right inferior pulmonary vein	185	0.807 [0.729-0.861]	1.31 [0.78-2.32]
Right superior pulmonary vein	140	0.795 [0.701-0.855]	1.39 [0.93-2.29]
Anterior papillary muscle	73	0.688 [0.539-0.778]	2.04 [1.37-3.15]
Posterior papillary muscle	33	0.709 [0.575-0.768]	1.73 [1.28-2.67]
Aortic cusps	163	0.828 [0.747-0.890]	0.81 [0.57-1.19]
Mitral valve	95	0.722 [0.650-0.792]	1.55 [1.02-2.58]
Left ventricle	274	0.822 [0.762-0.873]	3.27 [2.36-4.28]
Aortic valve	153	0.907 [0.875-0.928]	0.99 [0.77-1.38]

N refers to the number of evaluated cine-level annotated structure observations for each anatomic class. Dice similarity coefficient and 95th percentile Hausdorff distance (HD95) were calculated using frame-level measurements and summarized as median [IQR] by structure. HD95 is reported in millimeters.

Owing to the anatomical variability of smaller substructures, performance was lower but remained clinically meaningful. For the left atrial appendage, Dice median values were 0.79. For pulmonary vein segmentations, Dice coefficients were aggregated and achieved a median Dice of 0.78 (Figure 2). Papillary muscles, combining both anterolateral papillary muscle and posteromedial papillary muscle, achieved a Dice coefficient of 0.70, and aortic cusps achieved a Dice coefficient of 0.83 (Figure 3). The slightly lower performance for prediction of pulmonary veins, papillary muscles, and aortic cusps, as compared to the left atrial body, reflect the greater technical challenge of visualizing and segmenting these features. The aortic valve and mitral valve orifices achieved a Dice of 0.91 and 0.72, respectively.

### Hausdorff distance

Boundary accuracy was evaluated using the HD, with quantitative results provided in Table 2. For large structures such as the left atrium, Auto-Contour achieved minimal boundary error (HD 1.18 mm). The left atrial appendage demonstrated HD 1.81 mm, whereas pulmonary veins yielded an aggregated HD 1.44 mm. Valve structures and papillary muscles showed an aggregated HD of 1.89 and 1.27, respectively.

### Overall model performance

Across the test set, Auto-Contour consistently achieved Dice coefficients near or above 0.80 for most structures. Large chambers such as the left atrium surpassed 0.90, reflecting high agreement with human annotations. Smaller and more irregular structures such as the left atrial appendage and PV ostia achieved Dice values in the 0.79-0.78 range, similar to levels of interobserver variability reported in ultrasound segmentation studies. From the left ventricular region, the left ventricle achieved a Dice coefficient of 0.82 and the mitral valve and aortic valve achieved a Dice coefficient of 0.72 and 0.91, respectively. Smaller left ventricle regional structures, aortic cusps and papillary muscles achieved Dice scores of 0.83 and 0.70 respectively. When performance was suboptimal, the model still correctly localized the intended structure, with deviations limited to small edge discrepancies. This was reflected in narrow Dice SDs (0.05-0.10 across most structures), indicating consistent accuracy across diverse cases.

### Processing speed and prospective integration

Although not formally benchmarked in this retrospective analysis, Auto-Contour demonstrated near real-time performance on a modern graphics processing unit. Segmentation outputs were generated in <0.03 seconds per frame, sufficient for overlay on live ICE video at standard frame rate of 30 fps. In the first-in-human study, integration with the sensor-enabled ViewFlex X catheter and EnSite X mapping system demonstrated the feasibility of coupling AI-derived contours with catheter location data for 3-dimensional anatomic reconstruction and live mapping-system integration (Figure 4, Figure 5, Figure 6). This supports rapid, automated construction of 3-dimensional anatomical shells during procedures and represents an important step toward real-time AI-assisted mapping in the electrophysiology lab.

## Discussion

In this study, we demonstrated the feasibility of using Auto-Contour, a deep learning model to perform automated multistructure segmentation of ICE images. The model, trained on a multicenter data set of 5,496 annotated cine loops from 249 procedures, achieved high segmentation accuracy, including high agreement with human annotations of the left atrium (Dice coefficient 0.94) and left ventricle (Dice coefficient 0.82). Strong performance for smaller, more challenging structures, such as the left atrial appendage (Dice 0.79), the pulmonary vein ostia (Dice 0.78), papillary muscles (Dice 0.70), and aortic cusps (Dice 0.83). These findings demonstrate that deep learning methods can approximate expert-level ICE interpretation, even for anatomically variable and technically challenging structures, and that real-time performance (<0.03 seconds per frame) is achievable, supporting potential intraprocedural applications.

To our knowledge, this study represents one of the first comprehensive evaluations of AI for ICE imaging, a field that has lagged behind other imaging modalities in adoption of AI. The strong Dice coefficients we report, demonstrate performance approaching high agreement with expert annotations, a benchmark critical for clinical acceptance. For perspective, segmentation accuracies exceeding 0.9 are rarely reported for ultrasound-based imaging, due to ultrasound’s inherent noise, artifacts, and variable acquisition conditions. The ability of Auto-Contour to achieve this level of accuracy on ICE, which also has the additional technical complexity owing to catheter positioning, angulation, zoom, and variable gain settings, reflects both the effectiveness of the training strategy and the quality of the annotated data set. These results further suggest that deep neural networks can generalize effectively in the ICE domain when provided with enough high-quality labeled examples covering diverse scenarios.

Our findings build on and extend prior preliminary efforts by our group and others. Although general applications of AI in echocardiography (eg, automeasurements in transthoracic echo) have been reported, applications in ICE are newly emerging. Prior work on AI-assisted ICE has primarily focused on 3-dimensional anatomical reconstruction rather than frame-level, multistructure segmentation. Di Biase et al^18^ demonstrated the feasibility of reconstructing 3-dimensional left atrial shells, including veins and left atrial appendage, from ICE sweeps using the CartoSound FAM module, reporting strong correlations with computed tomography measurements, high operator satisfaction (mean qualitative score ∼4.3/5), and quick reconstruction times (∼65 seconds). However, their study did not evaluate frame-by-frame segmentation. Similarly, Akerström et al.^19^ assessed an early, precommercial CartoSound reconstruction tool and found that ∼69% of ablation lesion tags aligned within 3 mm of automatically reconstructed pulmonary vein antra, indicating reasonable anatomical accuracy; however, we note that the fluid load of the patient can change anatomical dimensions from computed tomography to day-of-procedure and therefore explain measurement discrepancy. Comparatively, Auto-Contour performs frame-by-frame, real-time semantic segmentation of multiple cardiac structures with quantitative validation, achieving median HD95 values ranging from 0.81 to 3.27 mm across annotated structures. These results complement and extend prior CartoSound-based approaches by introducing detailed, validated segmentation for both large and small structures, enabling procedural planning and live anatomical overlays. AI within the ICE workflow has potential patient clinical benefits, as indicated in a 2026 Europace study of 298 patients undergoing radiofrequency atrial fibrillation ablation, which found that AI-based left atrial reconstruction was associated with shorter total procedure time and left atrial dwell time, while maintaining similar acute success, safety, and 1-year freedom from atrial fibrillation recurrence compared with standard mapping.^20^ Another recent AI effort explores a system to guide image acquisition during ICE procedures. Huh et al^15^ proposed an AI that directs novice operators on how to adjust the ICE catheter to obtain standard views, using continuous closed-loop feedback. Such view-guidance AI is complementary to Auto-Contour and opens the possibility of future systems where AI assists operators in both acquiring optimal views and automatically interpreting those views by outlining critical anatomy. Together, these advancements point toward an AI-augmented workflow that could democratize ICE usage, reduce the learning curve for new operators, and provide decision support for experienced operators.

ICE has become an essential imaging modality in electrophysiology and structural heart, with prior work demonstrating its ability to improve outcomes and safety when used effectively.^21^ Field et al^21^ showed that ICE utilization during ventricular tachycardia ablation was associated with lower recurrence rates. These studies highlight both the power of ICE and the expertise required to use it optimally.

The introduction of Auto-Contour to the electrophysiology lab stands to enhance procedural accuracy, efficiency, and consistency. Automated anatomical contours allow electrophysiologists to verify anatomy without manually tracing or relying purely on mental visualization. For example, in a future variation of this approach being used during transseptal puncture, the AI can outline the fossa ovalis on ICE, helping ensure a safe puncture site away from the aortic root. Labeling pulmonary vein ostia during atrial fibrillation ablation could accelerate mapping and lesion placement. This holds the potential to reduce procedural time that would otherwise be spent switching to other imaging modalities or pulling in preacquired computed tomography scans. In these scenarios, Auto-Contour acts as a real-time second observer, offering standardized guidance for the primary operator.

Today, developing intraprocedural ICE-based anatomical shells relies on frame-by-frame manual anatomy tracing by a human annotator. The accuracy and robustness of the ICE-based anatomical shell is influenced by the number of segmentations that contribute to its surface, annotator experience, and anatomic variability. With Auto-Contour, the process can be largely automated: as the ICE catheter sweeps across the left atrium or left ventricle, the model’s segmented contours can be streamed directly into the mapping system, which builds them into a dynamic 3-dimensional model. Auto-Contour also provides immediate feedback and transparency to the physician and annotator, which may improve data collection efficiency and accuracy. A Dice of 0.94 for the left atrium and 0.82 for the left ventricle, Auto-Contour achieves accuracy comparable to expert contours and with real-time generation of these segmentations, this can significantly contribute to case efficiency and anatomical accuracy. Therefore, an integrated system that combines Auto-Contour with the location-registered position and orientation data of the ViewFlex X catheter (as in the ongoing first-in-human study) could allow the operator to obtain a full 3-dimensional left atrial model within minutes, without needing a preprocedural computed tomography, and thereupon sparing radiation and contrast exposure for the patient.

### Future directions

Looking forward, we aim to integrate the Auto-Contour model to run concurrently within a clinical workflow and test its impact in a prospective study. We will assess how this influences procedure metrics such as imaging time, total procedure time, fluoroscopy use, and perhaps physician stress or cognitive load. We would also gather user feedback from operators regarding the agreement of AI overlays. Another line of future enhancement is to incorporate 3-dimensional information and sensor data that may prove beneficial in more accurate 3-dimensional modeling of anatomy. Beyond segmentation, we posit other AI tasks that may complement Auto-Contour–such as detecting tool position (identifying the ablation catheter tip on ICE) or monitoring for complications (recognizing the echogenic cloud of a micro-bubble “steam pop”). The authors on this paper are actively involved in several such initiatives, harnessing their collective expertise in electrophysiology, imaging, and AI. We foresee a suite of AI tools that together make the electrophysiology lab of the future more intelligent and safer.

Finally, it is worth highlighting that although our focus was on intracardiac echo in the context of electrophysiology, primarily left atrial imaging for atrial fibrillation ablation, the developed techniques can be extended to other scenarios. ICE’s role in structural heart disease interventions, such as, guiding edge-to-edge mitral repair or atrial septal defect closures, can be augmented by an AI segmentation model that could be trained to label structures relevant in those procedures, such as the mitral valve, left ventricular outflow tract, among others. Given the modular nature of our pipeline, expanding the training data set to other structures would be feasible. The present work thus lays a foundation not only for atrial fibrillation ablation guidance but potentially a broader AI-assisted intracardiac imaging platform.

### Study Limitations

It is important to acknowledge the limitations of this study and the current model. First, the training and validation were performed on images from a single vendor’s ICE catheters (Abbott’s ViewFlex family). Although this ensured consistency in image characteristics, there may be some limitations in generalizing to images from other ICE systems (such as Siemens AcuNav). The model may require fine-tuning for different ultrasound probe characteristics. Second, because of the standardized imaging protocols, our evaluation does not consider view classification, which is relevant in a real-world setting and where image acquisition may be more varied. Third, our evaluation metric focused on Dice overlap and HD; this is a useful metric but does not capture all aspects of contour quality. In addition, the papillary muscles and aortic cusps were represented by relatively small evaluation counts; accordingly, performance estimates for these structures should be regarded as preliminary, since median Dice and HD95 values may be unstable and disproportionately influenced by a limited number of cases. Fourth, the segmentation was evaluated on 2-dimensional frames. In reality, ICE is a dynamic 2-dimensional imaging modality where visualizing a full 3-dimensional structure may require moving the catheter. Our model does not inherently enforce spatial consistency across frames; our approach instead segments each frame independently, so it is possible that slight frame-to-frame variations in the contour could occur. Smoothing algorithms or a 3-dimensional extension of the model could further improve stability. Fifth, all “ground truth” in this study is based on human expert annotations, which, although the best available standard, can themselves have variability. Although our data labeling protocol included adjudicating labels by senior annotators, we did not explicitly measure interobserver variability among our annotators; doing so would give further context to the AI’s performance. In future work, comparing the AI not only to single experts but to consensus or multiple experts could be informative. Furthermore, we did not compare the ICE-derived anatomical reconstructions to those acquired by computed tomography, which may offer a more objective comparison due to computed tomography’s deterministic imaging physics, as performed by Di Biase et al’s group.^18^

Another limitation is that our data set, although relatively large for an ICE study, is still modest by deep learning standards and includes a small amount of supraventricular tachycardia, ventricular tachycardia, and other electrophysiology procedures. Although this study was developed using a data set on the order of hundreds of imaging studies, by comparison, some AI applications in general echocardiography leverage tens or hundreds of thousands of studies.^12^ Despite the difficulty in accumulating ICE imaging data sets and the resultant relatively modest data set size, the model showed clinical-grade performance. As more data accrue (for example, as the ViewFlex X study continues to enroll (NCT06772493), we anticipate retraining and further improving the model’s robustness. Finally, because the present study relied on annotations generated by an internal labeling team, the interpretation of this validation study warrants appropriate criticism, and ongoing accrual of externally annotated data will support further validation.

## Conclusions

Auto-Contour demonstrated accurate, real-time, multistructure segmentation of ICE images during invasive cardiac procedures. By automating anatomic contouring, this approach may reduce operator dependence, improve procedural consistency, and support integration with 3-dimensional electroanatomic mapping systems. Future studies should evaluate its deployment in live clinical workflows, its impact on procedural efficiency and outcomes, and its potential to extend AI-assisted intraprocedural imaging, pending prospective clinical validation.

Perspectives **COMPETENCY IN MEDICAL KNOWLEDGE:** ICE is increasingly central to electrophysiology and structural heart interventions, yet interpretation remains operator-dependent and procedural views are not standardized. Auto-Contour demonstrated real-time, multistructure segmentation of ICE anatomy with high agreement for major cardiac chambers and acceptable performance for smaller, anatomically complex structures. Automated ICE segmentation may improve procedural consistency; support standardized anatomic recognition and facilitate integration with 3-dimensional electroanatomic mapping systems.

**TRANSLATIONAL OUTLOOK:** Prospective studies are needed to determine whether real-time integration of Auto-Contour into live ICE workflows improves imaging efficiency, reconstruction accuracy, fluoroscopy exposure, procedure time, operator workload, and clinical outcomes. Future work should evaluate performance across catheter platforms, institutions, and procedural settings, while refining 3-dimensional modeling and workflow-embedded AI guidance.

## Funding support and author disclosures

Funding for this research was provided by Abbott Laboratories. Drs Sokol, Sandler, Gilgur, and Elspas are employees of iCardio.ai Corporation and hold equity in the company; and these authors receive consulting fees and/or research support from industry entities, including Abbott Laboratories and GE Healthcare. Dr Gupta received grant support from 10.13039/100000050National Heart, Lung, and Blood Institute 1R56HL175516-01; is a cofounder of Heartbeat Health, Inc, a telehealth cardiology company, and iCardio.ai, an artificial intelligence echocardiography company; and has received fees for consulting and speaking engagements with Edwards LifeSciences and Boston Scientific. Drs Tanouye, Dahlen, and Dr Yao are employed by Abbott Laboratories. Buckland is the founder of CardioServ, a medical education and accreditation company; and holds equity in iCardio.ai. Dr Barbhaiya has received consulting fees from Abbott Laboratories and J&J MedTech. Dr Gopinathannair has received consulting fees from Abbott Laboratories, BSCI, J&J MedTech, and Sanofi; and a research grant from BSCI. Dr Hsu has received honoraria from Medtronic, Abbott, Boston Scientific, Biotronik, Janssen Pharmaceuticals, Bristol-Myers Squibb, Pfizer, Sanofi, Altathera Pharmaceuticals, Milestone Pharmaceuticals, Zoll Medical, iRhythm, Edwards Lifesciences, Atraverse Medical, Viz.ai, and Biosense-Webster; and has equity interest in Vektor Medical. All other authors have reported that they have no relationships relevant to the contents of this paper to disclose.
