# Supplementary material for: Accuracy of a Deep Learning Model in Intracardiac Echocardiography
Source: JACC Adv. 2026 Jun 15;5(7):102867. doi: 10.1016/j.jacadv.2026.102867 (PMC13285831; doi:10.1016/j.jacadv.2026.102867)
Supplement: Supplemental Material [file mmc1.docx]

**Supplementary Section**

**Supplementary Table 1. TRIPOD-AI Checklist**

| Section/Topic | Item | D/E | Checklist Item | Location in Manuscript | Notes / Gaps |
| --- | --- | --- | --- | --- | --- |
| TITLE |  |  |  |  |  |
| Title | 1 | D;E | Identify the study as developing or evaluating the performance of a multivariable prediction model, the target population, and the outcome to be predicted | Title: "Accuracy of a Deep Learning Model in Intracardiac Echocardiography" | Covered. Title identifies prediction model (deep learning), target population (ICE), and outcome (accuracy/segmentation). |
| ABSTRACT |  |  |  |  |  |
| Abstract | 2 | D;E | See TRIPOD+AI for Abstracts checklist | Abstract section | Abstract covers background, objective, methods, results, conclusions. Verify against separate TRIPOD+AI Abstracts checklist. |
| INTRODUCTION |  |  |  |  |  |
| Background | 3a | D;E | Explain the healthcare context and rationale for developing or evaluating the prediction model, including references to existing models | Background, ¶1–4 | Covered. Describes ICE clinical context, operator-dependence problem, prior AI work in echo, and gap in ICE AI. |
|  | 3b | D;E | Describe the target population and intended purpose in context of care pathway, including intended users | Background, ¶5–6 | Covered. Target = EP/SH procedures; users = electrophysiologists; purpose = automated segmentation for procedural guidance. |
|  | 3c | D;E | Describe any known health inequalities between sociodemographic groups | Table 1 | Diverse ethnic/racial groups included in study |
| Objectives | 4 | D;E | Specify the study objectives, including whether development or validation (or both) | Background, ¶6 ("In this study, the accuracy of Auto-Contour…is evaluated") | Covered. States both development and evaluation. Could be more explicit about "internal validation." |
| METHODS |  |  |  |  |  |
| Data | 5a | D;E | Describe the sources of data separately for development and evaluation datasets, rationale, and representativeness | Methods → Study Design and Data Sources, ¶1–2 | Covered. Two sources described (routine clinical ICE + First in Human study). 65/35 split noted. Representativeness partially addressed. |
|  | 5b | D;E | Specify dates of collected participant data, including start and end of accrual | Methods → Study Design and Data Sources | June 2024, and August, 2025 |
| Participants | 6a | D;E | Specify key elements of study setting including number and location of centres | Methods → Study Design and Data Sources, ¶2 ("Across participating high-volume EP centers…249 procedures") | Partially covered. "Multiple high-volume EP centers" but exact number of centers not stated explicitly. Affiliations list 7 sites. |
|  | 6b | D;E | Describe eligibility criteria for study participants | Methods → Study Design and Data Sources, ¶2 ("Cine loops that contained catheters in view were excluded") | Partially covered. Exclusion criterion stated. Inclusion criteria could be more explicit. |
|  | 6c | D;E | Give details of treatments received and how handled during model development, if relevant | Table 1 (Procedural Indications) | Covered. Procedure types listed. Treatment not directly relevant to segmentation task — reasonable to note briefly. |
| Data preparation | 7 | D;E | Describe data pre-processing and quality checking, including across sociodemographic groups | Methods → Image Annotation and Ground Truth, ¶2; Model Architecture, ¶1 ("resized to 256×256") | Partially covered. Cross-checking by second expert noted. No mention of QC across sociodemographic groups. |
| Outcome | 8a | D;E | Clearly define the outcome being predicted, time horizon, how/when assessed, rationale, consistency across groups | Methods → Image Annotation and Ground Truth; Evaluation Metrics | Covered. Outcome = pixel-wise segmentation labels. No time horizon (cross-sectional). Consistency across groups not discussed. |
|  | 8b | D;E | If outcome assessment requires subjective interpretation, describe qualifications of outcome assessors | Methods → Study Design, ¶2 ("A dozen ICE experts…minimum of five years") | Covered. Expert qualifications described. Demographic characteristics of assessors not reported. |
|  | 8c | D;E | Report any actions to blind assessment of outcome | Methods → Training, Validation, and Tuning | Separation of training and validation ensures segmentation data was blinded to model |
| Predictors | 9a | D | Describe choice of initial predictors and any pre-selection before model building | Methods → Model Architecture, ¶1 | Covered. Input = ICE frames (pixel data). Pre-trained on TTE segmentation task. No predictor selection in the traditional sense (image-in, segmentation-out). |
|  | 9b | D;E | Clearly define all predictors, including how and when measured | Methods → Image Annotation, ¶1–2; Model Architecture | Covered. Input is 256×256 ICE frames from DICOM cine loops. |
|  | 9c | D;E | If predictor measurement requires subjective interpretation, describe qualifications of assessors | N/A | Input is raw ultrasound imagery — no subjective predictor interpretation. |
| Sample size | 10 | D;E | Explain how study size was arrived at and justify sufficiency | Limitations, ¶2 | 249 studies used |
| Missing data | 11 | D;E | Describe how missing data were handled. Provide reasons for omitting data | Methods → Study Design, ¶2 (exclusion of catheters-in-view cines) | Partially covered. Exclusion criteria stated. No explicit discussion of missing annotations or incomplete cines. |
| Analytical methods | 12a | D | Describe how data were used for development and evaluation, including partitioning | Methods → Training, Validation, and Tuning, ¶1 ("≈80% training, 20% validation/testing…patient level") | Covered. |
|  | 12b | D | Describe how predictors were handled (functional form, rescaling, transformation, standardisation) | Methods → Model Architecture ("resized to 256×256") | Partially covered. Resize noted. Augmentations mentioned in abstract but not detailed in Methods body. |
|  | 12c | D | Specify type of model, rationale, all model-building steps including hyperparameter tuning, and method for internal validation | Methods → Model Architecture + Training, Validation, and Tuning | Covered. U-Net, GD Focal Loss, AdamW, early stopping, hyperparameter tuning via validation set. Could add more detail on augmentations. |
|  | 12d | D;E | Describe if/how heterogeneity in model performance was handled across clusters | Methods → Evaluation Metrics, ¶1 ("observations were clustered…") | Covered. Clustering acknowledged. Descriptive summaries used. No formal mixed-effects or cluster-level analysis. |
|  | 12e | D;E | Specify all measures and plots used to evaluate model performance | Methods → Evaluation Metrics (Dice, HD95); Table 3; Figure 1 | Covered. Dice and HD95 with IQR. No calibration/clinical utility measures, but these are less applicable to segmentation. |
|  | 12f | E | Describe any model updating from evaluation | N/A | No model updating performed. This is initial development + internal validation. |
|  | 12g | E | For evaluation, describe how model predictions were calculated | Methods → Model Architecture; Evaluation Metrics ("per-image processing time") | Partially covered. Inference described. Could specify hardware/GPU used for evaluation. |
| Class imbalance | 13 | D;E | If class imbalance methods were used, state why and how | Methods → Model Architecture ("Generalized Dice Focal Loss"); Image Annotation ("intra-cardiac cycle sampling for pulmonary veins") | Covered. GD Focal Loss addresses imbalance. Additional sampling for underrepresented structures noted. |
| Fairness | 14 | D;E | Describe any approaches to address model fairness and rationale | Table 1 | Diverse ethnic/racial groups included in study |
| Model output | 15 | D | Specify the output of the prediction model. Provide details for any classification and thresholds | Methods → Model Architecture ("pixel-wise class predictions"); Evaluation Metrics | Covered. Output = per-pixel class probabilities → segmentation masks. No explicit threshold discussion (argmax implied). |
| Training vs evaluation | 16 | D;E | Identify differences between development and evaluation data | Methods → Training, Validation, and Tuning | Partially covered. Patient-level split prevents leakage. No explicit comparison of train vs. test demographics/distributions. |
| Ethical approval | 17 | D;E | Name the IRB/ethics committee; describe consent or waiver | Methods → Study Design, ¶1 ("approved by the institutional review boards…informed consent was waived") | Covered. IRB approval and consent waiver stated. Specific IRB names not listed. |
| OPEN SCIENCE |  |  |  |  |  |
| Funding | 18a | D;E | Give source of funding and role of funders | Title page: "Funding for this research was provided by Abbott Laboratories" | Partially covered. Funder named. Role of funder not described (did Abbott influence design, analysis, or writing?). |
| Conflicts of interest | 18b | D;E | Declare conflicts of interest and financial disclosures | Title page: Declaration of competing interest | Covered. Detailed COI for all authors. |
| Protocol | 18c | D;E | Indicate where study protocol can be accessed, or state none prepared |  | None prepared - Data are not publicly available due to proprietary agreements |
| Registration | 18d | D;E | Provide registration info or state not registered | Methods → Study Design ("ClinicalTrials.gov NCT06772493") | Partially covered. First in Human trial registered. The retrospective AI study itself is not registered — state this explicitly. |
| Data sharing | 18e | D;E | Provide details of data availability |  | None prepared - Data are not publicly available due to proprietary agreements |
| Code sharing | 18f | D;E | Provide details of code availability |  | None prepared - Data are not publicly available due to proprietary agreements |
| PATIENT & PUBLIC INVOLVEMENT |  |  |  |  |  |
| Patient & Public Involvement | 19 | D;E | Provide details of PPI or state no involvement |  | No involvement |
| RESULTS |  |  |  |  |  |
| Participants | 20a | D;E | Describe flow of participants including number with/without outcome | Results, ¶1 ("5496 cine loops from 249 procedures"); Table 1 | Partially covered. No formal flow diagram. |
|  | 20b | D;E | Report characteristics overall and per data source, including demographics, sample size, missing data | Table 1 (demographics and indications) | Covered. Age, sex, race reported. Missing data amounts not reported. No breakdown by data source (clinical vs First in Human). |
|  | 20c | E | For evaluation, compare distribution of important predictors with development data | Table 1 (demographics and indications) | Partially covered. Age, sex, race reported. Missing data amounts not reported. No breakdown by data source (clinical vs First in Human). |
| Model development | 21 | D;E | Specify number of participants and outcome events in each analysis | Results, ¶1; Table 3 (counts per structure) | Partially covered. Total cines and segmentation counts given. |
| Model specification | 22 | D | Provide details of the full prediction model to allow predictions in new individuals | Methods → Model Architecture | Architecture described (U-Net, 256×256 input). No model weights, code, or API provided. Proprietary — state access restrictions explicitly. |
| Model performance | 23a | D;E | Report model performance with confidence intervals, including subgroups | Results → Segmentation Accuracy; Table 3 (Dice/HD95 with IQR) | Covered. IQR reported. No formal CIs (bootstrap or otherwise). No subgroup analysis by demographics. |
|  | 23b | D;E | If examined, report heterogeneity in model performance across clusters | NOT FORMALLY REPORTED | Mentioned in response letter that subgroup analyses by ICE view were exploratory and removed. State this in manuscript. |
| Model updating | 24 | E | Report results from any model updating | N/A | No model updating performed. |
| DISCUSSION |  |  |  |  |  |
| Interpretation | 25 | D;E | Give overall interpretation including issues of fairness | Discussion, ¶1–2 | Partially covered. Good interpretation of results and comparison to prior work. Fairness not discussed. |
| Limitations | 26 | D;E | Discuss limitations including non-representative sample, sample size, overfitting, missing data, generalizability | Limitations, ¶1–2 | Covered. Single vendor, no view classification, no inter-observer variability, modest dataset, internal validation. Well done. |
| Usability | 27a | D | Describe how poor quality or unavailable input data should be handled when implementing | Limitations, ¶1 (re: single vendor, varied gain settings) | We mention the input image requires acquisition by an experienced imaging specialist. |
|  | 27b | D | Specify whether users interact with input data or model, and expertise required | Discussion → Clinical Impact, ¶1–2 | Partially covered. Describes operator use case. Does not specify minimum expertise required to use the system. |
|  | 27c | D;E | Discuss next steps for future research re: applicability and generalizability | Future Directions, ¶1–2 | Covered. Prospective study, 3 dimensional integration, extension to structural heart, multi-vendor validation. |
